# Surgical Bacteriology

**Published:** 1891-11

**Authors:** 


SURGICAL BACTERIOLOGY. By N. SENN, M. D., Ph. D., Professor of Surgery in Rush Medical College, Chicago, and in the Chicago Polyclinic; Attending Surgeon to the Milwaukee Hospital; Consulting Surgeon to the Milwaukee County Hospital, and to the Milwaukee County Insane Asylum; Honorary Fellow College of Physicians of Philadelphia; Permanent Member of the German Congress of Surgeons; Corresponding Member Harveian Society, London; Honorary Member of La Academia de la Medicina de Mexico, etc. Second edition, thoroughly revised. Philadelphia: Lea Brothers & Co. 1891.

Whether the speedy exhaustion of the first edition of this work was due to the popularity of its author, or to the increasing interest in bacteriology, it is a pleasure to know that the subject has taken deep root among the medical men of America. Although not pursuing this line of work as actively as our foreign brethren, yet we are ready and willing to grasp and digest all that is prepared for us by those more favorably situated. Among the American bacteriologists, Professor Senn stands near the head, and his writings, therefore, possess weight and carry conviction. The volume before us is, strictly speaking, an exhaustive compend of the work done in recent years, and teems with extracts gathered from the writings of the ablest workers on the various subjects connected with bacteriology. Opinions and experiments at variance with each other are both presented, without any effort to harmonize them, except when the observations of the author, based upon original research, may tend to carry conviction to the one side or to the other. Even to those not especially interested in the study of microörganisms, the discussion in such chapters as The Hereditary Transmission of Microbic Diseases; Do Pathogenic Microörganisms Exist in the Healthy Body? Sources of Infection, etc., etc., must be of the greatest importance. Although more especially intended for the surgeon-pathologist, yet the general practitioner will find a full and free discussion of nearly all the microbic diseases with which he comes in contact. It is surprising the amount of literature mastered by Dr. Senn in the preparation of this work—the authors running into the hundreds. Twelve plates, illustrative of the various bacilli, embellish the book.

